# The Risk Factors Associated with Grip Lock Injuries in Artistic Gymnasts: A Systematic Review

**DOI:** 10.3390/ijerph20043589

**Published:** 2023-02-17

**Authors:** Erhan Kara, İsa Sağıroğlu, Hikmet Vurgun, Özgür Eken, Halil İbrahim Ceylan, Tomasz Gabrys, Magdalena Barasinska, Urszula Szmatlan-Gabrys, Peter Valach

**Affiliations:** 1 Coaching Education Department, Faculty of Sport Sciences, Tekirdag Namik Kemal University, Tekirdağ 59000, Turkey; 2 Kirkpinar Faculty of Sport Sciences, Trakya University, Edirne 22030, Turkey; 3 Coaching Education Department, Faculty of Sport Sciences, Manisa Celal Bayar University, Manisa 45040, Turkey; 4 Department of Physical Education and Sport Teaching, Inonu University, Malatya 44000, Turkey; 5 Physical Education of Sports Teaching, Faculty of Kazim Karabekir Education, Atatürk University, Erzurum 25030, Turkey; 6 Department of Physical Education and Sport, Faculty of Education, University of West Bohemia, 30100 Pilsen, Czech Republic; 7 Department of Health Sciences, Jan Dlugosz University, 42-200 Czestochowa, Poland; 8 Department of Anatomy, Faculty Rehabilitation, University of Physical Education, 31-571 Krakow, Poland

**Keywords:** artistic gymnastics, grip lock injury, wrist injury, forearm fracture

## Abstract

Artistic gymnastics (AG) is a sport that demands grace, strength, and flexibility, leading to a broad spectrum of injuries. The dowel grip (DG) is widely used by gymnasts to securely hold onto the high bar or uneven bars. However, incorrect usage of the DG can result in grip lock (GL) injuries. This systematic review aims to (1) identify studies that have investigated the risk factors related to GL injuries among gymnasts and (2) synthesize the key evidence. A comprehensive electronic search was conducted in the following databases: PubMed, ScienceDirect, Elsevier, SportDiscus, and Google Scholar, covering the period from their inception until November 2022. The data extraction and analysis were independently completed by two investigators. A total of 90 relevant studies were initially identified, out of which seven clinical trials met the eligibility criteria. For the quantitative synthesis, five studies were included. The details extracted from each article include: the sample characteristics (number, gender, age, and health status), the study design, the instrumentation or intervention used, and the final results. Our results revealed that the underlying causes of the risk factors of GL injuries were the irregular checking of the dowel grip and the mating surface of the bar, the tearing of the dowel of the leather strap, and the use of the dowel grip in different competition apparatuses. In addition, GL injuries may occur either as severe forearm fractures or mild injuries. Excessive flexion of the forearm and overpronation of the wrist during rotational movements, such as the swing or backward/forward giant circle, may increase the possibility of GL injury on the high bar. Future studies should focus on GL injury prevention strategy and rehabilitation protocol for GL injuries. Further high-quality research is required to establish the validity of these findings.

## 1. Introduction

Injuries are an unfortunate but common experience for many athletes. The number and type of injuries in each sport vary, depending on the physical and cognitive demands of the sport [1]. Artistic gymnastics (AG) is a competitive sport that involves a series of physical exercises performed on various apparatuses, including the floor, balance beam, uneven bars, and parallel bars. The goal is to perform these exercises with grace, poise, and control, executing movements such as flips, twists, and turns. Injuries in AG are common due to the physically demanding and high-flying nature of the sport. Some common injuries include sprains, strains, and fractures of the wrists, ankles, and knees, as well as cuts, bruises, and muscle strains. Repetitive motions and high-impact landings can also lead to overuse injuries, such as tendinitis and stress fractures. In addition, gymnasts are at risk of injury from falls and collisions, particularly on the balance beam and uneven bars [1,2,3]. In one study, Hootman et al. (2007) reported that AG has a particularly high rate of injury and ranks second among collegiate sports in injury rates during practice, surpassed only by spring football [4]. It is known that elite gymnasts often undergo intense training regimes, with many dedicating 20 or more hours per week for 48 weeks per year, and they suffer an average of 2.155 injuries per season, which can range from minor bruises, strains, and sprains to more serious fractures and dislocations [5]. Additionally, gymnasts can perform nearly year-round without adequate rest and recovery, which can make them more susceptible to overuse injuries, stress fractures, and other types of injuries, especially during periods of intense training [6]. 

A previous study reported that male gymnasts have a higher rate of upper extremity injuries (53.4%) compared to lower extremity injuries (32.8%) [6]. Sport specialization among young athletes has been linked to an increased risk of overuse injuries [7,8]. Gymnast’s wrist, also known as distal radius stress fracture, is a common overuse injury among gymnasts. This injury occurs when the wrist is subjected to repetitive loading and much twisting and bending at a young age, leading to a small crack in the bone. Exposing the still-maturing bones of early-aged gymnasts to high levels of stress or strain increases the risk of wrist injury [9,10]. Under normal conditions, the distal radius is at a greater risk of injury because it bears 80% of the axial loading during weight-bearing activities when the wrist is extended, while the ulna only bears 20% of the load. This makes the distal radius more vulnerable to fractures and other types of injuries [10,11]. The adequate reduction in and potential fracture remodeling of distal radio ulna fractures may be a concern in adolescents approaching skeletal maturity. Nonetheless, a previous study performed on twenty-five older children and adolescents noted that the treatment of displaced diaphyseal forearm fractures (radius–ulna) in gymnasts due to GL injuries could be effectively managed with closed reduction and casting, which can lead to satisfactory functional outcomes despite any residual angulation [12].

The dowel grip (DG) is a narrow cylindrical object which is commonly used among gymnasts, particularly in routines that involve hanging and support on an apparatus such as the high bar, rings, uneven bars, and parallel bars. The use of DG has several benefits, such as boosting grip strength and dexterity in the hands, forearms, and shoulders through the wrapping of the wrist and three middle fingers with a leather dowel, improving the gymnast’s ability to clench the bar with maneuverability, decreasing friction between the palm and bar, and enhancing muscular endurance and coordination in the upper body. As a result, the use of the dowel grip helps gymnasts maintain a secure hold on the bar and perform complex skills with greater ease and control [13,14,15]. 

However, poorly manufactured grips, grips that stretch and deteriorate with prolonged use, and the misuse of DG may lead to GL injuries, which was first scientifically coined by Samuelson et al. (1996) in the medical literature [15]. They reported that 82.6% of gymnasts who sustained a GL injury were found to have used dowel grips, and GL injuries had a tendency to occur due to the hyperpronation of the forearm and the internal rotation of the arm. This type of injury in male gymnasts often results from improperly winding the leather strap around the dowel during performance on the high bar and giant circles. Additionally, GL injuries occur when a gymnast’s grip fails and they are unable to maintain control of the equipment, potentially leading to physical injuries, extensor tendon injuries, and fractures of the hand, wrist, or forearm [13,16]. Additionally, these injuries pose a challenge as they require appropriate stabilization techniques for the broken arm before unlocking and removing the DG [10]. In such situations, it is extremely difficult to get the suspended gymnast down from the bar, and it is essential that two people hold the unstable position and carefully remove the DG from the gymnast’s hand [17]. When the gymnast’s body moves with momentum, this can lead to forearm fractures and acute extensor tendon injuries [10,15,16,17,18,19,20]. If a gymnast experiences such severe injuries, they may have to withdraw from competition without completing their routine [15,19,21]. Therefore, it is important to follow proper form and technique when using DG or any gymnastics equipment to reduce the risk of injury, and preventing GL injuries is considered a crucial factor in successful performance in AG. 

Furthermore, health problems in high-level athletes very often have their cause in training mistakes made at the beginning of their sports career [22]. This includes neglecting to maintain proper joint mobility as a result of poorly conducted strength training. In children and adolescents, the case reports of injuries related to strength training, including fractures of the growth plate and lower back injuries, are mainly due to misusing equipment or using inappropriate weights, improper technique, or a lack of qualified adult supervision [23,24]. Poor training affects the overall level of functional mobility in young athletes, a condition that is exacerbated in the later stages of their sports careers [23,25,26]. Many elements of artistic gymnastics are transferred to modern forms of movement related to dance or commonly-used fitness exercises [27]. Combining strength preparation with dance elements and those originating in artistic gymnastics can compound movement limitations, leading to injury through adolescence and growth spurts [6,28]. Therefore, it seems important to ensure the safety of popular recreational forms of movement to identify the most injury-prone areas of preparation. This paper fills a gap in understanding this issue. After all, professional sport is an area where seemingly imperceptible disruptions in the human musculoskeletal system most quickly lead to health issues. This is due to the application of heavy loads and the induction of states of overload on the musculoskeletal system [2,29,30]. Considering all this, although many coaches and gymnasts are aware of GL injuries, there is limited research on the risk factors associated with this type of injury. The aim of this review is to provide a comprehensive analysis of the literature and to identify the potential risk factors and underlying causes of GL injuries

## 2. Materials and Methods

**Figure 1:**
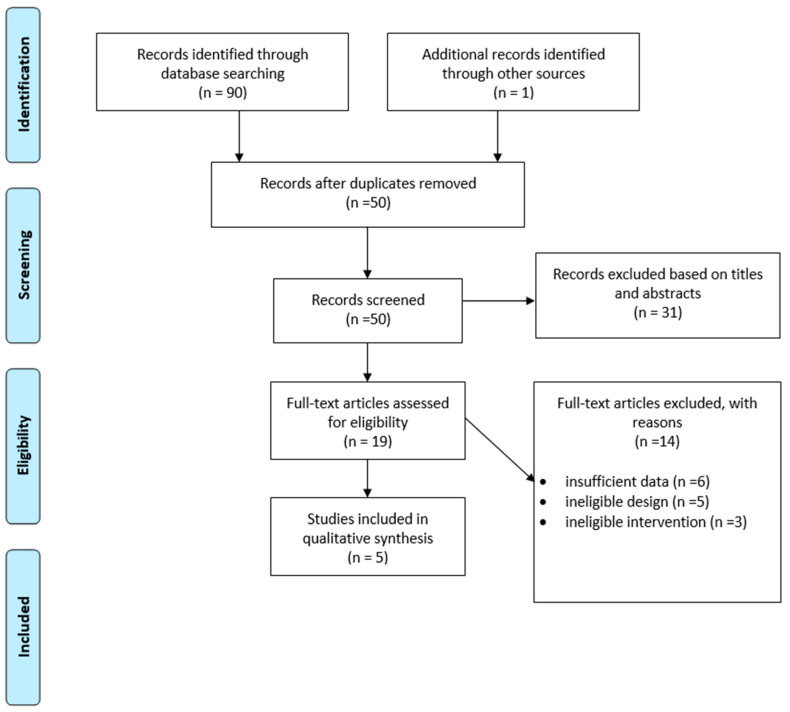
PRISMA flow diagram of the search strategy and study selection.

The present systematic review study followed the PRISMA (Preferred Reporting Items for Systematic Review and Meta-Analyses) guidelines for systematic reviews and meta-analyses [31] (Figure 1). Before conducting the search, a systematic review protocol was developed and registered with PROSPERO, the International Prospective Register of Systematic Reviews (http://www.crd.york.ac.uk/PROSPERO, accessed on 3 September 2022) (CRD42022355197).

### 2.1. Literature Search

The following search terms and MeSH terms were used in combination with keywords and Boolean operators: “male gymnasts”, “artistic gymnasts”, “high bar injury”, “injury mechanism in gymnastics”, “gymnastics and injury”, “grip lock injury”, “wrist injuries” OR “cases of radius and ulna bone fracture”, “gymnastics and forearm injuries”, “female gymnasts” AND “grip lock injury”, “injury prevention”, “treatment in artistic gymnastics” AND “healing”. The search results were organized in accordance with the IMRAD format [32] (Introduction, Methods, Results, Discussion) and 30 articles were analyzed. This systematic review was designed using the PICOS (Population, Intervention, Comparison, Outcome, Study Designs) search tool model (Table 1) and the Search Terms for each PICO Question (Table 2). 

### 2.2. Study Selection

Studies were included in this systematic review if they met the following criteria: (1) the participants were well-trained competitive artistic gymnasts, (2) the participants competed at the national, international, local, university, or regional level, (3) they were original research articles, (4) they underwent independent peer review, and (5) they were available in the English language. For the purposes of this review, an artistic gymnast was considered competitive if they participated in club (local), national, or international collegiate-level competitions. There were no age restrictions for the participants. Studies were excluded if they: (1) did not examine GL injuries, or examined those with a history of previous extremity injury or who performed gymnastics recreationally, (2) investigated other forms of gymnastics, such as rhythmic, aerobic, acrobatic, or trampoline gymnastics, (3) were non-English publications, or (4) were review articles or conference abstracts. Two authors independently screened the articles based on the inclusion and exclusion criteria, and full-text screening was performed by two authors (EK and IS) to resolve any disagreements by consensus. The reference lists of the included articles were searched for additional relevant studies, and authors were contacted for clarification if necessary. If no response was received, the article was excluded. There were no year limitations for this systematic review.

### 2.3. Data Extraction and Quality Assessment

To determine if there was consensus among the published expert opinions on the risk factors associated with GL injuries in artistic gymnasts and the mechanism of these injuries, two authors (EK and IS) independently reviewed the included papers and extracted relevant data whenever possible. Qualitative domains were created by the research team based on their review of the data extracted from the papers. No assessment of the methodological quality was conducted as part of this investigation. All of the included papers were expert opinion pieces (Evidence Level 5) and did not have any methodology that could be evaluated.

## 3. Results

### Search Results and Study Characteristics

The initial electronic database search resulted in the identification of 90 studies (as shown in Figure 1). After removing duplicates (*n* = 40), 50 studies underwent screening. Through the screening process, based on title, abstract, and eligibility, only five papers were found to be appropriate for inclusion in this systematic review (presented in Table 2). All five included papers were centered on GL injuries. The characteristics of the studies included in the systematic review can be found in Table 3. From the total number of ninety studies, five [13,18,20,21,34] that focused on GL injury were used for this review (Table 3). In contrast, other articles analyzed musculoskeletal issues, fractures of the distal radius, physical injuries in general, mechanisms of upper extremity injuries, muscle activity and its relation to chronic injury, and the impact of sport specialization. 

Regarding the findings of the synthesized five studies, including six cases, it was observed that four studies were conducted on male gymnasts and one study was conducted on female gymnasts. In the included studies, it was reported that GL injuries occurred during dismount, giant swing, giant loops, and hip circle maneuvers on the high bar (for male gymnasts) and uneven bars (for female gymnast). Moreover, these injuries typically resulted in open both-bone forearm fractures, physeal injuries, extensor tendon ruptures, and fractures of the hand, wrist, or forearm. It was determined that the most common injury is a stretching of the musculotendinous junction of the EDC/IF/MF/RF with elongation. Additionally, in the studies, it was observed that different methods were selected for the treatment of GL injuries, including operative, non-operative, conservative, closed reduction, and cast immobilization. In some studies, the time period for gymnasts to return to training or competition after GL injuries ranged from a minimum of 8 weeks to a maximum of 2 years, while in some studies, it is not specified whether they returned to competition or not.

## 4. Discussion

In this systematic review, the risk factors associated with GL injuries in artistic gymnasts were determined through an analysis of studies in the scientific literature. The analysis showed that this paper is the first systematic review to investigate GL injury cases, filling a significant gap in the literature by identifying the associations between risk factors and GL injuries in artistic gymnasts. The findings of the current study, in short, show that the main cause of GL injuries is the hyperpronation position of the wrist. In addition, the condition of the DG and improper strapping and improper cubital grip position are common risk factors for GL injuries in artistic gymnasts

GL injuries occur in many forms of movement in which the exerciser uses equipment. Many forms of sports involve very young people. Their musculoskeletal apparatus is not sufficiently strengthened for the demands of the sport [24]. Additionally, in some of the cases used for analysis, the ossification process has not been completed, as indicated by the age of the subjects [35,36]. The observed effect of body weight on the incidence of injury is an important indication for the construction of programs taking this factor into account [23,37]. The analysis of the research indicates the need to take into account two factors in the efforts commonly used in amateur and youth sports: the level of biological maturation and body weight [38,39]. Observations made in other studies [40,41] indicate that this problem affects many sports eagerly practiced in by children. These include ice hockey (strain during stick handling) [42,43], tennis [44], badminton [45], squash (racket handling) [46,47], rowing and kayaking (paddle work) [48,49], and sport climbing [50]. Excessive strain in this segment of the musculoskeletal system is, therefore, a common cause of injury. It is, therefore, becoming a health problem commonly accompanying children’s physical activity. In artistic gymnastics, it is most common due to a combination of two factors: young age (people who are not biologically mature) and the need to overcome a relatively heavy load during exercise, which is body weight [51]. A similar situation occurs in rock climbing, which is a young discipline commonly practiced by people of all ages [50,52]. It should be assumed that the problem there will soon arise and will have a mass character. The experience of gymnastics points to the need for preventive measures in this form [53,54]. 

A previous study showed that using GL results in a wrist load of 4.5 times the body weight. The combined effects of increased forces and rotational speeds put stress on growth plates and distal radioulnar joints, leading to an increased risk of both acute trauma and overuse syndromes [55]. However, the management of GL injuries varies based on the individual’s condition, and the surgical treatment of forearm fractures may involve a range of both conservative and non-conservative methods [56]. A study reported that fractures in the distal ulna region, which include both bones, were the third most common type of forearm injury in the pediatric population, accounting for 40% of all fractures [57]. It has been reported that gymnasts with reduced range of motion (ROM) [11] who have a GL injury in their wrists may experience pain; despite this, they may return to training prematurely [11]. Given that injuries specific to artistic gymnastics are related to mechanical stress, any injury prevention strategies can potentially reduce the frequency and severity of GL injuries [58]. Elite gymnasts often undergo intensive training, which can be around 30–50 h a week [59]. Thus, mental and physical fatigue may increase the risk of acute injuries such as GL [60]. 

When the included studies were analyzed, cubital grip position was the most common position in the course of GL injury. The cubital grip position occurs when the dowel of the leather strap meets the bar during forearm pronation, wrist flexion, and internal rotation of the shoulder [21]. Regarding the included studies, it was observed that only four studies were conducted on males (four studies, five cases). Another previous study performed by Yong-Hing et al. [25] and DiFiori et al. [31] reported the physical injury of a 13-year-old right-hand-dominant male gymnast due to tensile force during a swing on the high bar and the dowel of DG. Under normal circumstances, this is likely to produce stress on growth plates and distal radioulnar joints as a result of the force generated by the forearm during routines performed on high bar, rings, and uneven bars [55]. Another case study conducted on a male gymnast by Sathyendra and Payatakes [20] indicated that the gymnast experienced a serious extensor tendon injury and a non-displaced fracture of the ulnar styloid. Additionally, they observed that the GL injury had a negative impact on various muscle–tendon units, such as intramuscular and intratendinous stretching, decreased contractility, reduced elasticity, and increased susceptibility to peritendinous adhesion, based on intraoperative findings [20]. According to the aforementioned studies, gymnastics coaches should be familiar with the high-risk cubital grip position [16], which has a high risk of GL injury, and should also observe the gymnasts frequently for the correct use of this technique. The GL management and rehabilitation process of a gymnasts may be essential in reaching the level of pre-injury performance. Moreover, nutrition may also play an essential role in recovery during the rehabilitation period. However, there have been no data reported stating whether nutrition affects GL injury cases.

Moreover, for clinic evaluation of GL injuries, it is necessary to perform radiography, whereas magnetic resonance imaging is essential for determining the severity of the damage in a tendon [18]. Considering the included studies in the present study, in a male two-case study conducted by Bezek et al. [18], it was reported that during the physical examination of a gymnast taken to the orthopedics clinic with superficial palm abrasions, ecchymosis, and dorsal edema on the wrist after 24 h of injury, it was found that the gymnast suffered from an extensor tendon injury and an ulnar styloid fracture and he was treated without surgery. Nevertheless, a limitation at an angle of 60° in the metacarpophalangeal joint of the index finger was determined. For the second patient, the gymnast suffered from ruptured extensor tendons along with open fractures of the radius and ulna, and was treated surgically. However, at 2.5 years post-injury, the final outcome of the study observed residual extension delay in the index finger, long finger, and ring finger, as well as a 45° loss in wrist flexion [18]. Additionally, another case report carried out by Updegrove et al. (2015) reported that a 15-year-old male gymnast suffered a segmental forearm fracture involving the growth plate due to a grip lock injury. It was observed that there was pain in the second and third metacarpal bones, wrist, and forearm distal of the gymnast due to GL, yet no pain in the elbow; however, it was determined during radiography that there were diaphyseal fractures in the radius and ulna, as well as an avulsion injury on the third metacarpal pad [13]. 

In our study, regarding the studies related to female gymnasts, a recent study found that, among adolescent girls, the majority of injuries (foot and ankle sprains or strains, 51%) occurred in the lower extremity, followed by upper extremity injuries (30.8%), torso/spine injuries (13%), and head/neck injuries (0.8%) [61]. Another study on pediatric gymnastics revealed that young athletes, particularly female athletes, were prone to wrist and lower arm fractures and upper extremity injuries in childhood rather than adolescence. Furthermore, both male and female adolescents had an increase in the occurrence of concussions, as well as foot and ankle injuries [62]. 

In the literature, as far as we know, a single case study (*n* = 1) was identified for DG injury in female gymnasts. According to this case study, which is presented in the current study, Colon et al. [21] reported that a 13-year-old female pediatric gymnast suffered a diaphyseal fracture of both bones in her forearm as a result of a GL injury. Additionally, they reported that the injury was treated successfully with closed reduction and cast immobilization. Furthermore, during the two-year follow-up, the patient (she) did not report any pain, and was actively performing gymnastics with normal grip strength and range of motion [21]. No reports exist on GL injuries in female gymnasts, except for the above study. Previous studies suggest that female gymnasts have a lower risk of GL injuries due to their lack of dowel grips and infrequent exposure to high-force drill routines. The likely reason for this is that, despite male and female gymnasts producing similar force transfers on high bars and uneven bars, the diameter of the uneven bars does not fully fit and lock into the circumferences of skin grips, compared to high bars [21,56]. Moreover, the other reason is that the gripping technique of the two apparatuses is different; female gymnasts have smaller hands than male gymnasts, and their body weight is less than that of male gymnasts, decreasing the likelihood of GL injuries in female gymnasts. Gymnasts usually use the same DG over a period of a couple of months or years due to reasons such as comfort, superstition, or economy. However, worn out, stretched, or dated DG may cause the gymnast to become susceptible to GL injuries [13,18,21]. Another study, published by Samuelson et al. [15], showed that 38 high school coaches reported 17 injury cases, whereas 32 university coaches reported 21 GL-based injury cases. While 20 of these injuries were stated to be forearm fractures, the rest were reported to be sprains or minor injuries. The same researchers attributed the above-mentioned GL injuries to excessively large, worn, or stretched dowel grips (eighteen gymnasts), slipping of the grips over the wrists (eight gymnasts), and some technical errors (seven gymnasts). As a different mechanism related to the causes of GL, gymnasts may experience sweating during training or competition, which could lead to moisture in the dowel grips. Constant exposure to moisture can cause the DG to wear out and decrease their lifespan, increasing the risk of injury. Therefore, it is crucial to store the dowel grips in a dry environment after training or competition and for gymnastics coaches to regularly check the athletes’ dowel grips. In total, when all the included studies were reviewed, it was found that there is no rehabilitation protocol describing GL injuries for athletes receiving treatment [21].

Our study has some limitations. In our study, the risk factors associated with GL injuries in artistic gymnasts were determined through a comprehensive analysis of the available scientific literature. The main limitation of the presented study is its type. It is a study based on the analysis of literature data, which limits the number of cases considered, the measurement methodology, and the training solutions used in the athletes under investigation. For this reason, the results of the study must be interpreted with caution. The results can be extrapolated to other sports on the basis of hypotheses requiring confirmation. The strength of the presented study is the separation of risk factors for injury during GL. The weakness is the impossibility of transferring these results at a high level of detail to other disciplines in which GL occurs. The present study only determined means for studies that reported the number of gymnasts and injury rates. A single case of GL injury (*n* = 1) was found in a female gymnast [21], but it is unclear if there were any recurring cases of GL injury in the same arm. To identify future cases of GL injuries, cooperation with the International Gymnastics Federation and gymnastics committees of various countries should be established. This systematic review analysis found that GL injuries in artistic gymnasts can vary from minor injuries to serious forearm fractures, leading to withdrawal from training and, potentially, performance loss. Regular maintenance and inspection of dowel grips is necessary to ensure proper fit and to check for signs of wear. If signs of improper fit or wear are detected, such as stretching, stitch weakness, or tearing, the use of the dowel grip should be stopped immediately [13]. Furthermore, GL injuries caused by DG can be avoided through proper grip fit, regular maintenance of grips, limiting their use, and educating athletes, trainers, and coaches [18]. 

Lastly, the results of the analyses presented in the paper provide a rationale for experimental studies linking GL strengthening to the response to increased loads in gymnasts. They also allow, based on the results obtained, to design ba-tests in other sports; this applies to disciplines in which the risk of injury during GL occurs. Previous knowledge indicates that one way to prevent GL injuries is to reduce load. In many sports, especially those where very young athletes compete, this is impossible. This is because the load is due to the weight of their body or the weight of the equipment. Additionally, in general sports, GL loads are high, especially in tennis, climbing, and kayaking. A practical recommendation is the constant functional control of mobility in the wrist joint and observation of GL soreness. Since it has been found that the main cause of GL injuries is the hyperpronation of the wrist, it is necessary to control the condition of equipment and the technique of performing exercises in which this position occurs.

## 5. Conclusions

Based on all the analyses, it was found that the main cause of GL injuries is the hyperpronation of the wrist. Unfortunately, this position may lead to the dowel of the leather strap being caught and locked around the bar. In addition, the irregular checking of the mating surface of the bar and the DG by gymnasts, the dowel of the leather strap being torn, the same DG being used in different competition apparatuses, the DG not being worn correctly, the DG being worn out due to abrasion and not being replaced with a new one, the gymnast’s preference of a non-fitting DG, and the DG sliding up the wrist and therefore leading it to become excessively loose were determined to be the risk factors and underlying causes of GL injuries. Gymnastics coaches should be made aware of the first intervention during GL injury. It is thought that this may be important in terms of GL injury prevention strategy.

## Figures and Tables

**Table 1:** PICOS Tool in Study Design.

P	Well-trained competitive gymnasts of any age and gender.
I	Operative, non-operative, conservative, closed reduction, and cast immobilization methods.
C	Does not apply.
O	Extensor indicis proprius muscle, distal radial–ulnar joint crepitus, joint range of motion, and immobilization.
S	No restrictions regarding study design.

**Table 2:** Search Strategy.

PICOS Question 1 Search Terms	Boolean Operator		PICOS Question 2 Search Terms Search Terms
Grip lock injury (MeSH) [33] Wrist injury (MeSH) [33] OR	AND/OR	P	Female gymnast (MeSH) [33] OR Male gymnast (MeSH)
Forearm fracture OR Metacarpal fracture (MeSH) [33]	AND/OR	I	Splints (forearm) (MeSH) [33]
Operative non-operative		C	Does not apply
Radioulnar joint (MeSH) [33] OR Diaphyseal fracture radius/ulna OR Radius/ulna, avulsion (MeSH) [33]		O	High bars injury OR injury mechanism gymnast Treatment in AG (MeSH) [33]
	These terms were used as inclusion criteria and were not limited to searching PubMed, as only a limited number of systematic reviews and guidelines were found using just the P, I, and C terms.		

Abbreviations: MeSH, Medical Subject Heading (database); PICO, patient problem or population, intervention, comparison, and outcome(s) or study designs. AG: artistic gymnastics.

**Table 3:** Characteristics of included trials.

References	Age/Sex	Level	Mechanism	Injury	Treatment	Outcome	Return to Practices/Play/Competition
Bezek et al. [18]	20 M	Division I	On high bar, overgrip position during dismount	Ulnar stiloid avulsion; EDC strain at musculotendinous junction, PQ strain	Short arm/ elbow cast, 4 weeks	35° EIP lag, DRUJ crepitus	5 months
	18 M	High school senior	On high bar, overgrip position during dismount	Open both-bone forearm fracture; complete rupture IF EDC musculotendinous junction: stretching EDC/IF/MF/RF with elongation	Operative	MCP extension lac IF/MF/RF, extension contractures digit/wrist; 45° loss wrist flexion	Not stated
Sathyendra and Payatakes, [20]	24 M	College	On high bar, overhand grip during giant swing	Nondisplaced ulnar styloid fx; rupture musculotendinous junction EDC; adhesions EIP and IF EDC to compartment floor; intratendinous attenuation extensors to IF/MF/RF	Operative	Extensor lag 60° IF and MF, 35°	Return with some apprehension
Updegrove et al., 2015 [13]	15 M	Not stated	On high bar, during giants	Salter Harris II Radius fracture, diaphyseal fracture radius/ulna, avulsion base	Operative	Full return to function	Not stated
Yong-Hing et al., [34]	13 M	National	On high bar, during giants loops	Distal radial physeal injury	Palm-to-elbow plaster cast, 4 weeks	Not stated	8 weeks
Colon et al., [21]	13 F	Regional	On uneven bar during swing (hip circle)	Diaphyseal both-bone forearm fracture	6 weeks, cast/short forearm brace	Full return to function	2 years

Abbreviations: EDC: Extensor digitorum communis; EIP: Extensor indicis proprius; IF/MF/RF: Index finger/middle finger/ring finger; MC: Metacarpal; MCP: Metacarpophalangeal joint; PQ: Pronator quadratus; M: Male; F: Female

## Data Availability

Not applicable.

## References

[1] Sweeney E.A., Howell D.R., James D.A., Potter M.N., Provance A.J. (2018). Returning to Sport After Gymnastics Injuries. Curr. Sport. Med. Rep..

[2] Moeskops S., Oliver J.L., Read P.J., Cronin J.B., Myer G.D., Lloyd R.S. (2019). The Physiological Demands of Youth Artistic Gymnastics: Applications to Strength and Conditioning. Strength Cond. J..

[3] Campbell R.A., Bradshaw E.J., Ball N.B., Pease D.L., Spratford W. (2019). Injury epidemiology and risk factors in competitive artistic gymnasts: A systematic review. Br. J. Sport. Med..

[4] Hootman J.M., Dick R., Agel J. (2007). Epidemiology of collegiate injuries for 15 sports: Summary and recommendations for injury prevention initiatives. J. Athl. Train..

[5] Saluan P., Styron J., Ackley J.F., Prinzbach A., Billow D. (2015). Injury Types and Incidence Rates in Precollegiate Female Gymnasts. Orthop. J. Sport. Med..

[6] Caine D.J., Maffulli N. (2005). Epidemiology of Children’s Individual Sports Injuries. Epidemiology of Pediatric Sports Injuries.

[7] Hernandez M.I., Biese K.M., Schaefer D.A., Post E.G., Bell D.R., Brooks M.A. (2021). Different Perceptions of Parents and Children on Factors Influencing Sport Specialization. J. Sport Rehabil..

[8] Burwell M., DiSanti J., Valovich McLeod T.C. (2022). Early Sport Specialization in College Athletes and the Impact on Health-Related Quality of Life: A Critically Appraised Topic. J. Sport Rehabil..

[9] Watkins R.A., De Borja C., Ramirez F. (2022). Common Upper Extremity Injuries in Pediatric Athletes. Curr. Rev. Musculoskelet. Med..

[10] Hart E., Meehan W.P., Bae D.S., D’Hemecourt P., Stracciolini A. (2018). The Young Injured Gymnast. Curr. Sport. Med. Rep..

[11] Wolf M.R., Avery D., Wolf J.M. (2017). Upper Extremity Injuries in Gymnasts. Hand Clin..

[12] Zionts L.E., Zalavras C.G., Gerhardt M.B. (2005). Closed Treatment of Displaced Diaphyseal Both-Bone Forearm Fractures in Older Children and Adolescents. J. Pediatr. Orthop..

[13] Updegrove G.F., Aiyer A.A., Fortuna K.L. (2015). Segmental Forearm Fracture Due to Grip-Lock Injury in Male Gymnast. JBJS Case Connect..

[14] Handoll H.H., Madhok R., Howe T.E., Handoll H.H. (2006). Rehabilitation for distal radial fractures in adults. Cochrane Database of Systematic Reviews.

[15] Samuelson M., Reider B., Weiss D. (1996). Grip Lock Injuries to the Forearm in Male Gymnasts. Am. J. Sports Med..

[16] Tabila E.V., Kahanov L. (2008). Grip Lock: A Unique Mechanism of Injury in Gymnastics. Athl. Ther. Today.

[17] Hecht S.S., Burton M.S. (2009). Medical Coverage of Gymnastics Competitions. Curr. Sport. Med. Rep..

[18] Bezek E.M., VanHeest A.E., Hutchinson D.T. (2009). Grip Lock Injury in Male Gymnasts. Sport. Health A Multidiscip. Approach.

[19] Gabel G.T. (1998). Gymnastic Wrist Injuries. Clin. Sport. Med..

[20] Sathyendra V., Payatakes A. (2013). Grip Lock Injury Resulting in Extensor Tendon Pseudorupture: Case Report. J. Hand Surg. Am..

[21] Colon R., Olivella G., Pinci M., Rivera C., Ramírez N., Guzmán H. (2021). Diaphyseal Both-Bone Forearm Fracture Due to a Grip Lock Injury in a Female Pediatric Gymnast. JBJS Case Connect..

[22] Hamlin M.J., Wilkes D., Elliot C.A., Lizamore C.A., Kathiravel Y. (2019). Monitoring Training Loads and Perceived Stress in Young Elite University Athletes. Front. Physiol..

[23] Dahab K.S., McCambridge T.M. (2009). Strength Training in Children and Adolescents: Raising the Bar for Young Athletes?. Sport. Health Multidiscip. Approach.

[24] Faigenbaum A.D., Myer G.D. (2010). Resistance training among young athletes: Safety, efficacy and injury prevention effects. Br. J. Sports Med..

[25] Malm C., Jakobsson J., Isaksson A. (2019). Physical Activity and Sports—Real Health Benefits: A Review with Insight into the Public Health of Sweden. Sports.

[26] Myer G.D., Lloyd R.S., Brent J.L., Faigenbaum A.D. (2013). How Young Is Too Young to Start Training?. ACSMs. Health Fit. J..

[27] Russo L., Palermi S., Dhahbi W., Kalinski S.D., Bragazzi N.L., Padulo J. (2021). Selected components of physical fitness in rhythmic and artistic youth gymnast. Sport Sci. Health.

[28] Phillips C. (1999). Strength training of dancers during the adolescent growth spurt. J. Danc. Med. Sci..

[29] Aicale R., Tarantino D., Maffulli N. (2018). Overuse injuries in sport: A comprehensive overview. J. Orthop. Surg. Res..

[30] Orejel Bustos A., Belluscio V., Camomilla V., Lucangeli L., Rizzo F., Sciarra T., Martelli F., Giacomozzi C. (2021). Overuse-Related Injuries of the Musculoskeletal System: Systematic Review and Quantitative Synthesis of Injuries, Locations, Risk Factors and Assessment Techniques. Sensors.

[31] Liberati A., Altman D.G., Tetzlaff J., Mulrow C., Gøtzsche P.C., Ioannidis J.P.A., Clarke M., Devereaux P.J., Kleijnen J., Moher D. (2009). The PRISMA statement for reporting systematic reviews and meta-analyses of studies that evaluate health care interventions: Explanation and elaboration. J. Clin. Epidemiol..

[32] Sforza C., Eid L., Ferrario V.F. (2000). Sensorial Afferents and Center of Foot Pressure in Blind and Sighted Adults. J. Vis. Impair. Blind..

[33] MeSH Database: Pubmed (Website). https://www.nlm.nih.gov/mesh/meshhome.html.

[34] Yong-Hing K., Wedge J.H., Bowen C. (1988). V Chronic injury to the distal ulnar and radial growth plates in an adolescent gymnast. A case report. J. Bone Joint Surg. Am..

[35] Cavallo F., Mohn A., Chiarelli F., Giannini C. (2021). Evaluation of Bone Age in Children: A Mini-Review. Front. Pediatr..

[36] Mirtz T. (2011). The Effects of Physical Activity on the Epiphyseal Growth Plates: A Review of the Literature on Normal Physiology and Clinical Implications. J. Clin. Med. Res..

[37] McQuilliam S.J., Clark D.R., Erskine R.M., Brownlee T.E. (2020). Free-Weight Resistance Training in Youth Athletes: A Narrative Review. Sport. Med..

[38] Almeida-Neto P.F.D., de Medeiros R.C.D.S.C., de Matos D.G., Baxter-Jones A.D., Aidar F.J., de Assis G.G., Silva Dantas P.M., Cabral B.G.D.A.T. (2021). Lean mass and biological maturation as predictors of muscle power and strength performance in young athletes. PLoS ONE.

[39] Albaladejo-Saura M., Vaquero-Cristóbal R., González-Gálvez N., Esparza-Ros F. (2021). Relationship between Biological Maturation, Physical Fitness, and Kinanthropometric Variables of Young Athletes: A Systematic Review and Meta-Analysis. Int. J. Environ. Res. Public Health.

[40] DiFiori J.P., Caine D.J., Malina R.M. (2006). Wrist Pain, Distal Radial Physeal Injury, and Ulnar Variance in the Young Gymnast. Am. J. Sports Med..

[41] DiFiori J.P. (1999). Overuse Injuries in Children and Adolescents. Phys. Sportsmed..

[42] Donskov A.S., Humphreys D., Dickey J.P. (2019). What Is Injury in Ice Hockey: An Integrative Literature Review on Injury Rates, Injury Definition, and Athlete Exposure in Men’s Elite Ice Hockey. Sports.

[43] Benson B.W., Meeuwisse W.H. (2005). Ice Hockey Injuries. Epidemiology of Pediatric Sports Injuries.

[44] Bylak J., Hutchinson M.R. (1998). Common Sports Injuries in Young Tennis Players. Sport. Med..

[45] Goh S.L., Mokhtar A.H., Mohamad Ali M.R. (2013). Badminton injuries in youth competitive players. J. Sports Med. Phys. Fitness.

[46] Horsley I.G., O’Donnell V., Leeder J. (2020). The epidemiology of injuries in English professional squash; A retrospective analysis between 2004 and 2015. Phys. Ther. Sport.

[47] Horobeanu C., Johnson A., Pullinger S.A. (2019). The Prevalence of Musculoskeletal Injuries in Junior Elite Squash Players. Asian J. Sports Med..

[48] Baugh C.M., Kerr Z.Y. (2016). High School Rowing Injuries: National Athletic Treatment, Injury and Outcomes Network (NATION). J. Athl. Train..

[49] Isorna-Folgar M., Leirós-Rodríguez R., Paz-Dobarro R., García-Soidán J.L. (2021). Injuries Associated with the Practice of Calm Water Kayaking in the Canoeing Modality. J. Clin. Med..

[50] Meyers R.N., Hobbs S.L., Howell D.R., Provance A.J. (2020). Are Adolescent Climbers Aware of the Most Common Youth Climbing Injury and Safe Training Practices?. Int. J. Environ. Res. Public Health.

[51] Patel T.S., McGregor A., Williams K., Cumming S.P., Williams S. (2021). The influence of growth and training loads on injury risk in competitive trampoline gymnasts. J. Sport. Sci..

[52] Morrison A.B., Schoffl V.R. (2007). Physiological responses to rock climbing in young climbers. Br. J. Sports Med..

[53] Armstrong R., Relph N. (2021). Screening Tools as a Predictor of Injury in Gymnastics: Systematic Literature Review. Sport. Med.—Open.

[54] Daly R.M. (2001). Balancing the risk of injury to gymnasts: How effective are the counter measures?. Br. J. Sports Med..

[55] Neal R.J., Kippers V., Plooy D., Forwood M.R. (1995). The influence of hand guards on forces and muscle activity during giant swings on the high bar. Med. Sci. Sport. Exerc..

[56] Concannon L.G., Loveless M.S., Matsuwaka S.T., Sweeney E. (2020). Upper Extremity Injuries in Gymnasts.

[57] Vopat M.L., Kane P.M., Christino M.A., Truntzer J., McClure P., Katarincic J., Vopat B.G. (2014). Treatment of diaphyseal forearm fractures in children. Orthop. Rev..

[58] Hume P.A., Bradshaw E.J., Brueggemann G.-P. (2013). Biomechanics: Injury Mechanisms and Risk Factors. Gymnastics.

[59] Weiker G.G. (1989). Musculoskeletal problems and the gymnast. Adv. Sport. Med. Fit..

[60] Pettrone F.A., Ricciardelli E. (1987). Gymnastic injuries: The Virginia experience 1982-1983. Am. J. Sport. Med..

[61] Thomas R.E., Thomas B.C. (2019). A systematic review of injuries in gymnastics. Phys. Sportsmed..

[62] Tisano B., Zynda A.J., Ellis H.B., Wilson P.L. (2022). Epidemiology of Pediatric Gymnastics Injuries Reported in US Emergency Departments: Sex- and Age-Based Injury Patterns. Orthop. J. Sport. Med..

